# Use of Municipal Solid Waste Incinerator Fly Ash in Coal Fly Ash-Based Geopolymer Matrix: Mechanical Performance and Heavy Metals Immobilization

**DOI:** 10.3390/ma19173730

**Published:** 2026-09-01

**Authors:** Zehua Zhao, Yi Wang, Dapeng Zhang, Linli Liu, Houhu Zhang, Ganghua Pan, Zhicheng Zhu

**Affiliations:** 1 School of Materials Science and Engineering, Southeast University, Nanjing 211189, China; zhaozehua@nies.org; 2 Nanjing Institute of Environmental Sciences, Ministry of Ecology and Environment of the People’s Republic of China, Nanjing 210042, China; 3 East China Regional Technology Center of Hazardous Waste Environmental Risk Prevention and Control, Nanjing 210032, China

**Keywords:** MSWIFA, CFA, geopolymer, Ca(OH)_2_, heavy metals

## Abstract

**Highlights:**

- The addition of MSWIFA reduced the compressive strength and fluidity of geo-polymer
- Moderate Ca(OH)_2_ enhanced the compressive strength but reduced the fluidity
- The leaching toxicity of heavy metals greatly decreased after geopolymerization

**Abstract:**

Municipal solid waste incineration fly ash (MSWIFA) poses environmental risks due to high chloride and heavy metal contents. This study solidified MSWIFA via geopolymerization with coal fly ash (CFA), using low-temperature calcination and water washing to remove dioxins and chlorides, respectively. All formulations effectively immobilized heavy metals below regulatory limits. Increasing addition of calcined-washed fly ash (CWFA) from 0% to 25% reduced the 28 d compressive strength from 32.5 MPa to 4.8 MPa and fluidity from 185 mm to 128 mm, while increasing the content and modulus of alkali activator both enhanced these properties. The incorporation of moderate Ca(OH)_2_ promoted the substitution of Na^+^ by Ca^2+^ within the gel framework under the combined action of the alkaline activator, thereby favoring the formation of gel network. When the dosage of Ca(OH)_2_ increased to 7.5%, the 28 d compressive strength increased from 45.75 MPa to 53.33 MPa. However, excessive Ca(OH)_2_ resulted in more unreacted residues and accelerated carbonation, which occupied active sites and disrupted gel continuity, leading to a slight decrease in strength to 49.85 MPa. This work provided a viable pathway for the utilization of MSWIFA into geopolymer, especially for optimizing Ca regulation to modify compressive strength, fluidity, and heavy metals toxicity.

## 1. Introduction

Accelerated urbanization and improved living standards have intensified the burden of municipal solid waste treatment. MSWI has been widely adopted as a primary disposal route in China for its volume reduction, treatment capacity, and energy recovery potential. However, the incineration process produces substantial amounts of MSWIFA, which is commonly enriched in chlorides, heavy metals, and dioxins [1]. The composition of MSWIFA is highly dependent on the heterogeneity of municipal waste streams across regions [2,3]. Furthermore, incineration technology will also significantly influence the MSWIFA composition. For instance, as the dominant technology in China, grate furnaces produce MSWIFA typically enriched in chlorides, calcium, and alkali metals. In contrast, fluidized-bed incinerators generate MSWIFA that is comparatively richer in amorphous aluminosilicates [3,4]. Such compositional variability often leads to inconsistent performance under identical stabilization or utilization conditions.

The current MSWIFA treatment strategies mainly involve vitrification/sintering, cement-based solidification, and stabilization/solidification processes [5,6]. Although vitrification can effectively densify MSWIFA and yield aggregates for construction applications, the high cost of energy and treatment of secondary fly ash cannot be ignored [7,8]. In comparison, sintering consumes less energy but cannot effectively reduce the waste volume. The stabilization/solidification technologies can immobilize MSWIFA within dense matrices, thereby limiting the migration of hazardous constituents such as heavy metals and dioxins and reducing associated environmental risks [9]. Conventional cement-based solidification is often hindered by significant volume expansion, inadequate mechanical strength, and limited long-term stability [10]. In contrast, geopolymerization enables the effective encapsulation or chemical binding of heavy metals within compact aluminosilicate networks, yielding environmentally benign MSWIFA-based products [11]. In addition, geopolymer-based solidification typically offers higher compressive strength, lower heavy metal leachability, and superior durability [12]. Therefore, it has attracted growing global interest as a promising strategy for the safe treatment and resource utilization of various solid wastes.

Compared with conventional precursors such as CFA and ground granulated blast furnace slag, MSWIFA contains certain levels of Ca and chlorides, but fewer Si/Al components. Consequently, introducing MSWIFA into alkali-activated systems presents both resource utilization potential and notable uncertainties regarding performance and environmental safety. Liu et al. prepared alkali-activated cementitious materials using MSWIFA and metakaolin, reporting that the partial substitution of 10% metakaolin by MSWIFA enhanced the 28 d and 90 d compressive strengths by nearly 200%, which was attributed to the increased availability of reactive aluminosilicates, reaction degree, and structural integrity [13]. Fan et al. demonstrated that the geopolymer matrix in CFA-based geopolymers and ordinary Portland cement offered superior stabilization efficiency for the heavy metals in MSWIFA [14]. Liu et al. developed alkali-activated materials using MSWIFA and steel slag, showing that steel slag not only supplied additional Ca sources but also improved mechanical strength and heavy metal immobilization [15]. Cao et al. investigated co-geopolymerization of MSWIFA and CFA and confirmed satisfactory environmental safety and long-term stability of the composite system [16]. Sai et al. further constructed an alkali-activated matrix using MSWIFA and yellow phosphorus slag, achieving high immobilization efficiencies for Zn, Pb, and Cd, with retention rates reaching 99.9%, 99.4%, and 99.8% after 60 d of leaching, respectively [17].

While physical pretreatment offers a straightforward means to reduce chloride loading, its efficiency in enhancing the mechanical performance of MSWIFA-based binders remains marginal. The excessive presence of heavy metals and chlorides in MSWIFA disrupts the aluminosilicate framework, hindering the formation of a three-dimensional aluminosilicate network. This interference ultimately retards cement hydration and leads to reduced compressive strength [18]. For instance, Bui Viet et al. reported a marginal strength gain in mortar incorporating 30% MSWIFA, achieving 20 MPa after chloride removal via washing [19]. Similarly, Chen et al. observed nearly a 30% increase in compressive strength when utilizing washed MSWIFA, while the absolute 28 d strength remained modest at 16.4 MPa [20]. Lu et al. further corroborated that substituting 10–30% cement with NH_4_Cl-washed MSWIFA promoted hydration kinetics [21]. Nevertheless, the overall enhancement imparted by water washing on MSWIFA-based cementitious properties is rather limited. Although water or acid washing effectively reduces chloride loading, it exhibits negligible removal efficiency for refractory dioxins [22]. Given the stringent regulatory limits on dioxin content in geopolymer-based construction materials, conventional pretreatment alone is insufficient to meet the environmental safety requirements for MSWIFA utilization. This necessitates the development of integrated pretreatment technologies capable of simultaneous dechlorination and dioxin destruction.

In alkali-activated materials, Ca is a critical factor governing reaction kinetics, gel composition, and final performance [23]. It is well established that systems with less Ca content predominantly yield highly cross-linked N-A-S-H gels. Conversely, increasing Ca content typically leads to the formation of Ca-bearing gels, such as C-(N)-A-S-H or C-A-S-H, significantly altering reaction rates, pore structures, and mechanical properties [24,25]. Particularly under high-alkalinity conditions, Ca incorporation not only accelerates the dissolution of aluminosilicate precursors and early-stage gel precipitation, but also modifies the local coordination environments of Si/Al and the connectivity of the gel framework, thereby affecting densification and strength development [25]. While studies suggest that moderate Ca(OH)_2_ addition will enhance reaction kinetics and refine microstructure, excessive Ca can result in residual unreacted phases, increased carbonation, and limited network polymerization [26]. However, most existing research focuses on conventional systems (e.g., metakaolin or slag), and systematic insights into the regulatory role of Ca in MSWIFA-based alkali activation remain limited [27,28,29]. Specifically, the optimal extent of calcium removal during washing and whether post-washing calcium supplementation is required to balance reaction activity and gel polymerization lacks theoretical guidance.

Accordingly, this study presents a systematic investigation covering the low-temperature calcination, water washing, and geopolymerization. Furthermore, Ca(OH)_2_ was introduced to regulate the reaction, and the underlying mechanism governing the influence of Ca on the alkali activation process was elucidated.

## 2. Materials and Methods

### 2.1. Raw Materials and Reagents

The MSWIFA was collected from a waste-to-energy plant in Jiangsu Province, China, and CFA was obtained from a coal-fired power plant in Henan Province. The main chemical compositions of the two materials are summarized in Table 1. The sodium silicate solution (water glass, SiO_2_-27.98%, Na_2_O-8.53%, and H_2_O-63.49%) used in this study was supplied by Yourui Refractory Materials Co., Ltd. JiaXing, China in Zhejiang Province, with a modulus of 3.3. The used NaOH was analytical reagent grade, with purity ≥ 98%.

### 2.2. Calcination and Washing of MSWIFA

The MSWIFA was ground and sieved through a 25-mesh. Then it was dried at 105 °C for 12 h to constant weight. A 15 g portion of the dried sample was placed in an inert corundum boat and calcined in a tubular furnace at 300~500 °C for 1 h. Dioxin concentrations were determined before and after thermal treatment. Assuming that dioxins are predominantly destroyed rather than volatilized during low-temperature calcination, and the mass loss of MSWIFA is negligible, the TEQ-based removal efficiency was calculated from the ratio of TEQ concentrations before and after treatment. The calcined MSWIFA was then subjected to water-washing pretreatment to remove soluble salts. Washing tests were conducted at liquid-to-solid ratios of 4, 6, 8, and 10 mL/g (50 min), with durations of 10~90 min (at 20 min intervals, 10 mL/g) under stirring at 300 rpm. Each condition was repeated for four consecutive washing cycles (10 mL/g and 50 min). After each cycle, solid-liquid separation was carried out, and the chloride concentration in the supernatant was analyzed to assess chloride removal performance. The final solid product after calcination and washing was labeled as CWFA.

### 2.3. Preparation of Geopolymer Experiment

A series of composite alkali activators with varying moduli were formulated by adding a 10 mol/L NaOH solution to water glass. Afterwards, the alkali activators were aged at room temperature for 24 h prior to use. The modulus of the sodium silicate solution was defined as the molar ratio of SiO_2_ to Na_2_O (n(SiO_2_/Na_2_O)). The water-to-binder ratio was defined as the mass ratio of exterior water and solid materials (W/B). The raw materials were weighed according to the mix proportions and dry-mixed in a planetary mixer for 1 min to ensure homogeneous particle distribution. The pre-prepared alkali activator was then added, and the mixture was stirred following a three-stage procedure: slow stirring for 2 min, a 15 s pause during which the polycarboxylate-based superplasticizer was added at a dosage of 1.5 wt.% by mass of the total solid precursors, and fast stirring for 2 min. The fresh geopolymer paste was cast into 40 mm × 40 mm × 40 mm cubic molds in three equal layers, with each layer vibrated for 1 min on a vibrating table to expel entrapped air. After surface leveling, the molds were covered with plastic film to minimize moisture evaporation and cured at 50 °C in a rapid cement curing chamber for 1 d before demolding. The demolded specimens were subsequently transferred to a standard curing chamber maintained at 20 ± 1 °C and 95% relative humidity until the specified curing time, at which point macroscopic and microscopic tests were conducted. The effects of CWFA content, alkali activator dosage, activator modulus, and Ca(OH)_2_ addition on the macro-properties of the geopolymers were systematically investigated. In addition, the heavy metal leaching toxicity of the geopolymer specimens was evaluated in accordance with the Chinese standard Solid Waste Leaching Toxicity-Acetic Acid Buffer Solution Extraction Procedure HJ/T 300-2007 [30]. The used solution was prepared by diluting 17.25 mL of glacial acetic acid to 1 L, yielding a pH of 2.64 ± 0.05. The extraction was conducted at a liquid-to-solid ratio of 20 mL/g (extractant volume to dry sample mass) using an end-over-end tumbler operating at 30 ± 2 r/min under ambient temperature for 18 ± 2 h. Detailed mixture proportions and preparation parameters for all samples are summarized in Table 2. The water-to-binder (W/B) ratio was adjusted within 0.33–0.47 to compensate for the increased water demand of MSWIFA and Ca(OH)_2_, thereby ensuring comparable flowability.

### 2.4. Compressive Strength and Fluidity Test

The cubic specimen surfaces in contact with the testing machine platens were polished flat to ensure uniform stress distribution during compression. The loading was controlled by displacement, and the rate was 0.5 mm/min. Three identical specimens were prepared and tested under the same conditions. The final compressive strength reported for the mix ratio is taken as the arithmetic average of the three measured values. The fluidity and fluidity loss of fresh slurry were determined with reference to Methods for testing uniformity of concrete admixtures GB/T 8077-2023 [31]. The truncated cone circular die (upper diameter 36 mm, lower diameter 60 mm, height 60 mm) was placed on a horizontal smooth glass plate, the fresh pure slurry was injected into the truncated cone circular die, the mold was lifted vertically and started to be timed, so that the slurry flowed naturally for 30 s. The two vertical diameters of the slurry were measured with a ruler, and the average diameter was taken as the fluidity value.

### 2.5. Characterization

The composition of solid raw materials was detected by X-ray fluorescence (XRF, Thermo-Fisher, Waltham, MA, USA, 3600). To determine the main crystalline phase of each product, the X-ray powder diffractometer (XRD, Billerica, MA, USA, CuKα, λ_Cu_ = 1.54 A, Bruker D8 ADVANCE) was employed with the scanning angle 2θ ranging from 5° to 90° at a rate of 10 °/min. The microscopic morphology and elements distribution of samples were investigated by scanning electron microscopy (SEM, ZEISS, Oberkochen, Germany, SU8010). Before testing, the representative fragments were broken from the interior of the cured geopolymer specimens and dried at 60 °C for 24 h. The samples were mounted on aluminum stubs with conductive carbon tape and sputter-coated with a thin Au layer (~10 nm) using an ion sputter coater (target: 99.99% Au; Ar atmosphere; ~15–20 mA; 40–60 s) to ensure electrical conductivity. The functional groups were obtained by the Fourier transform infrared spectra (FTIR, Thermo Fisher Nicolet Is5, Thermo Fisher Scientific Inc., Waltham, MA, USA) in the range of 4000–400 cm^−1^. The contents of heavy metals were detected by the inductively coupled plasma optical emission spectrometry (ICP-OES, Agilent, Santa Clara, CA, USA, 5110). For this instrument, a value of 0.01 mg/L is considered the practical quantification threshold, and results above this level are regarded as reliable and quantitatively meaningful. The toxic organic pollutants in MSWIFA were measured according to the Chinese standard of HJ 77.3-2008 Determination of polychlorinated dibenzo-p-dioxins (PCDDs) and polychlorinated dibenzofurans (PCDFs) in solid waste through isotope dilution HRGC-HRMS (JMS-800D, Tokyo, Japan) [32]. For each condition, three parallel tests were conducted to ensure experimental accuracy. The Origin 2018 was used to draw data graphs. The standard deviations of the detected data were calculated using the STDEV.P function in Excel (Office 2019) and shown by the error bars in the figures. Unless otherwise specified, all percentages reported in this manuscript refer to mass fraction.

## 3. Results and Discussion

### 3.1. Characterization of CFA, MSWIFA, and CWFA

**Figure 1:**
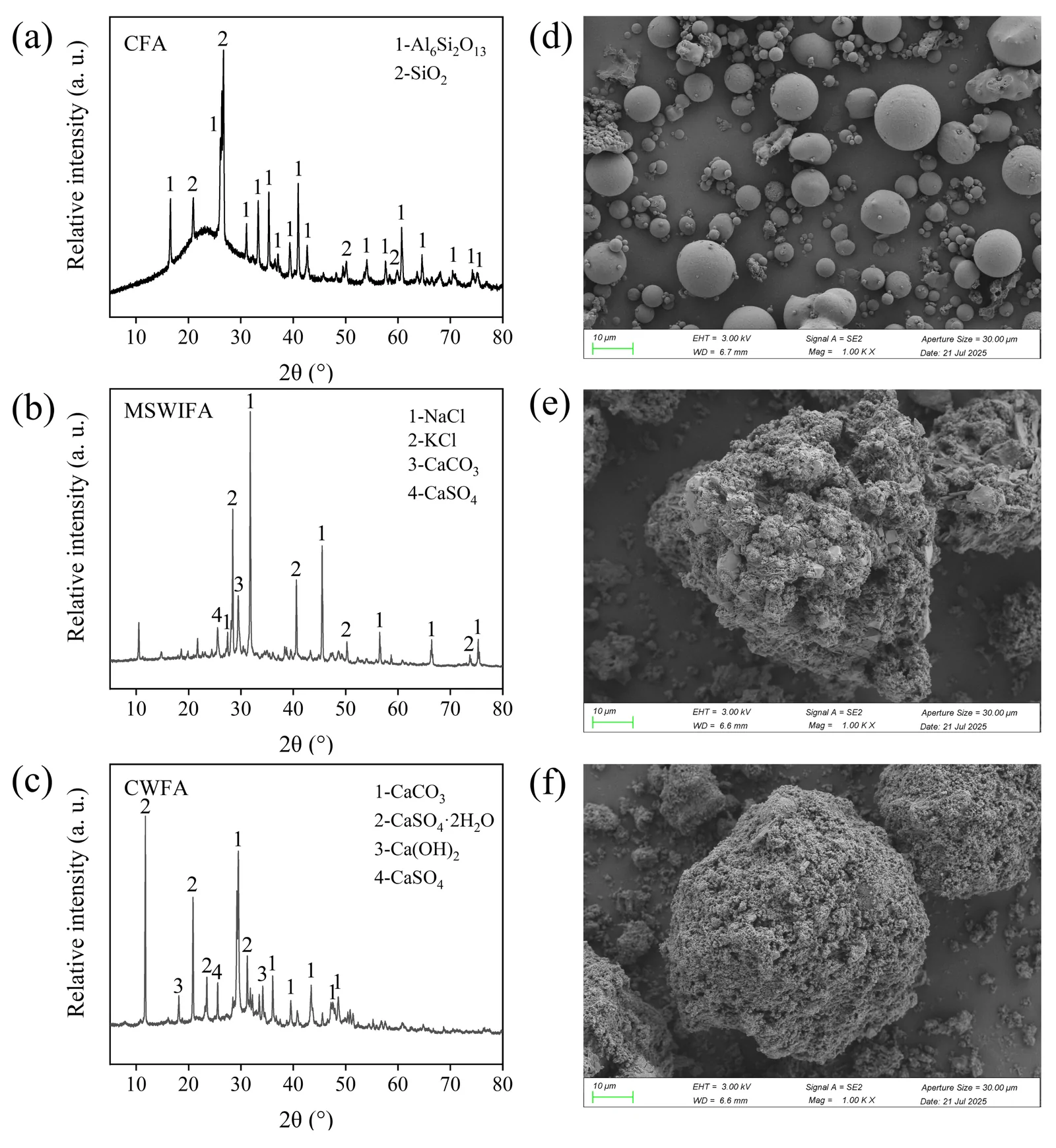
Mineral phase and surface morphology of CFA (**a**,**d**), MSWIFA (**b**,**e**), and CWFA (**c**,**f**).

The MSWIFA is characterized by high contents of CaO (33.52%) and Cl (20.50%). While the high Ca content favors the formation of Ca-containing gels or hydration-like products during the geopolymerization [33], the excessive chloride salts compromise structural stability and adversely affect paste fluidity and long-term durability [34]. Therefore, a water-washing pretreatment is typically required prior to resource utilization. In contrast, CFA represents a typical low-Ca content (3.33%) precursor but contains high levels of SiO_2_ (61.79%) and Al_2_O_3_ (28.71%), which can serve as the critical precursors for constructing the geopolymer backbone [35]. The XRD patterns and morphological observations in Figure 1 further revealed distinct microstructural differences. The quantitative determination of the total amorphous content was not performed in this study. Accurate quantification would require a well-defined fully crystalline internal standard and rigorous background modeling; these requirements are difficult to meet in complex, multi-phase systems such as MSWIFA and geopolymers, where the reliability of such measurements is inherently limited. CFA is primarily composed of Al_6_Si_2_O_13_ and SiO_2_, exhibiting predominantly regular spherical particles with smooth surfaces. Conversely, MSWIFA mainly consists of NaCl, KCl, CaCO_3_, and minor CaSO_4_. The MSWIFA particles are morphologically complex, featuring rough surfaces, porous structures, flocculent aggregates, and severe agglomeration, with some surfaces covered by acicular or rod-like crystals. This intricate texture can provide abundant reactive interfaces for alkali activation. After water washing at 8 mL/g, 70 min, and 3 cycles, the soluble NaCl and KCl phases were largely removed, leaving CaCO_3_ as the dominant residual crystalline phase of CWFA. During the subsequent hydration and drying process, a portion of CaSO_4_ transformed into CaSO_4_·2H_2_O, accompanied by the formation of trace amounts of Ca(OH)_2_. Moreover, the removal of surface-adhered salts exposed previously blocked pores and channels, thereby facilitating the penetration of the alkaline solution and subsequent polycondensation reactions.

### 3.2. Dioxin Removal and Desalination Performance

**Figure 2:**
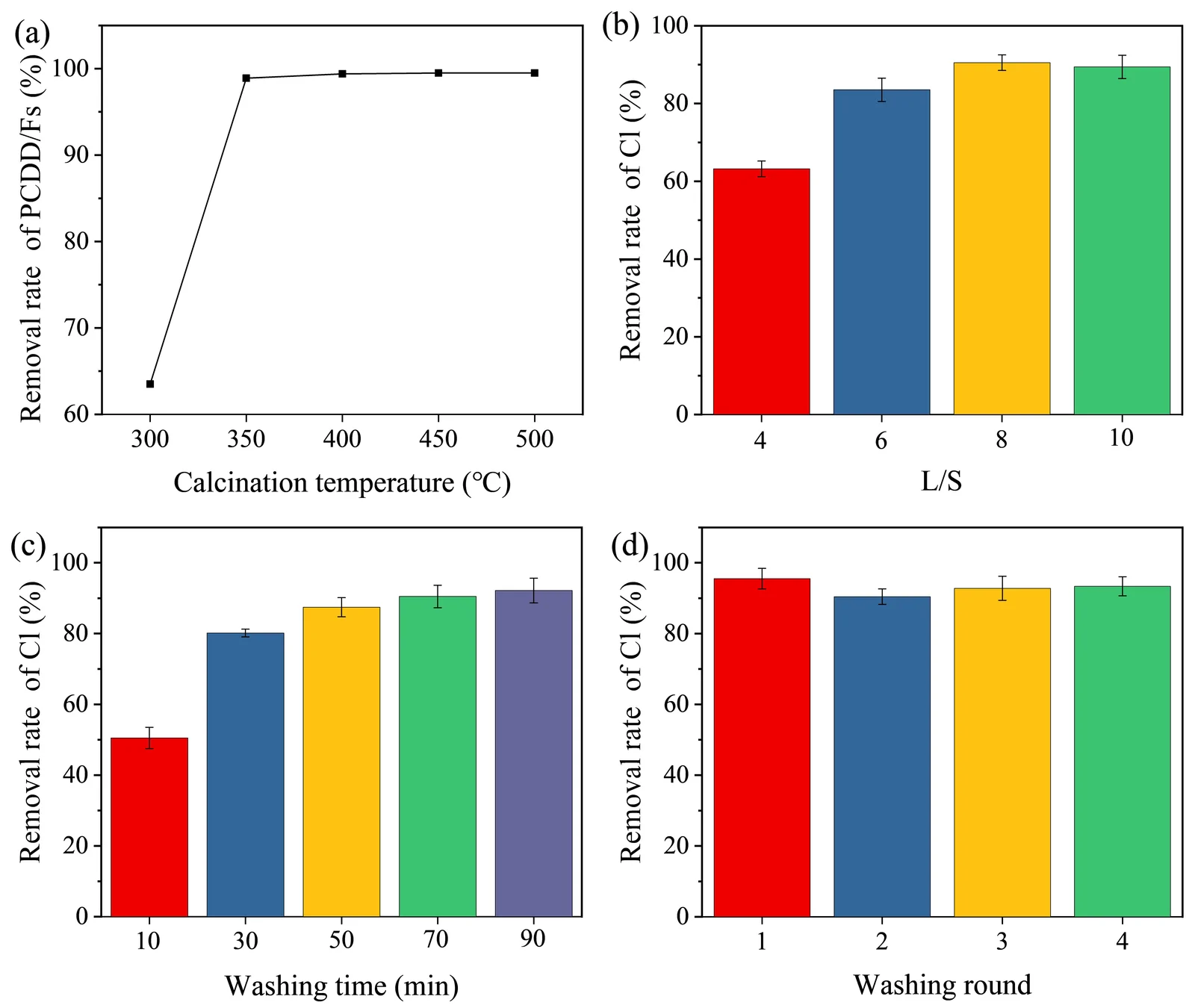
Removal of PCDD/Fs with different calcination (**a**) and Cl with different washing conditions (**b**–**d**).

To reduce the pollutant toxicity of MSWIFA and improve the sample preparation, the low-temperature calcination was employed to decompose dioxins, followed by the water washing for salt removal. The raw MSWIFA sample exhibited a polychlorinated dibenzo-p-dioxins and dibenzofurans (PCDD/Fs) concentration of 1349 ng/kg, with a toxic equivalent (TEQ) of 77.52 ng TEQ/kg. After calcination at 300 °C for 1 h, the PCDD/Fs concentration decreased to 269.2 ng/kg, and the TEQ dropped to 28.23 ng TEQ/kg, achieving a TEQ-removal efficiency of 63.58%. When the temperature was further increased to 350 °C, the PCDD/Fs concentration and TEQ significantly decreased to 8.53 ng/kg and 0.84 ng TEQ/kg, respectively, with the TEQ-removal efficiency rapidly increasing to 98.92%. As shown in Figure 2, although the TEQ values over 300 °C all complied with the threshold of 50 ng TEQ/kg stipulated by the Technical specification for pollution control of fly-ash from municipal solid waste incineration HJ 1134-2020 [36] for resource utilization, the dioxin removal efficiency at 350 °C exhibited a significant enhancement compared to that at 300 °C. Moreover, further elevating the temperature yielded negligible gains in dioxin removal, stabilizing the TEQ-removal efficiency at approximately 99.5%, which was consistent with some previous studies [37,38]. Therefore, 350 °C was selected as the optimal calcination temperature to balance treatment efficiency and energy consumption. 

The effects of the liquid-to-solid ratio (L/S), washing time, and washing rounds on the Cl removal rate were also studied. As Figure 2 shown, all three factors exhibited a typical rapid initial increase followed by stabilization trend. As the L/S ratio increased from 4 to 8, the Cl removal rate rose sharply from 63% to 90%. However, when the L/S further increased to 10, the Cl removal rate almost did not change, suggesting that readily soluble chlorides were largely exhausted during the initial stage, and the Friedel’s salt in the MSWIFA was hard to be extracted by the water washing [39]. Therefore, the further increase in L/S did not significantly improve the efficiency of de-chlorination. The influence of the washing time on the Cl removal rate was similar to that of the liquid–solid ratio. It also showed a trend of being fast at the beginning and then slowing down. This indicated that the soluble chlorides such as NaCl and KCl on the surface and in the open pores of the MSWIFA can quickly enter the liquid phase in the early stage of water washing. As the washing process continues, the residual chlorides gradually transform into a slow-release part that is constrained by internal diffusion and local structure. Therefore, the improvement in dechlorination efficiency slowed down significantly. Moreover, multi-stage washing demonstrated a notable cumulative effect, with the removal rate reaching approximately 85% after a single wash and increasing to 94% after three washes, albeit with diminishing returns per cycle. Based on a comprehensive evaluation of de-chlorination performance and process economy, an L/S ratio of 8, a washing time of 70 min, and three washing cycles were determined as the optimal pre-treatment parameters before the geopolymer preparation, with the product labeled as CWFA. This regime effectively removed the majority of dioxins and soluble chlorides while maintaining reasonable water consumption and operational costs. The CWFA obtained under this condition was used to prepare geopolymer in the following work.

### 3.3. Factors on the Geopolymer Properties

#### 3.3.1. Content of CWFA

**Figure 3:**
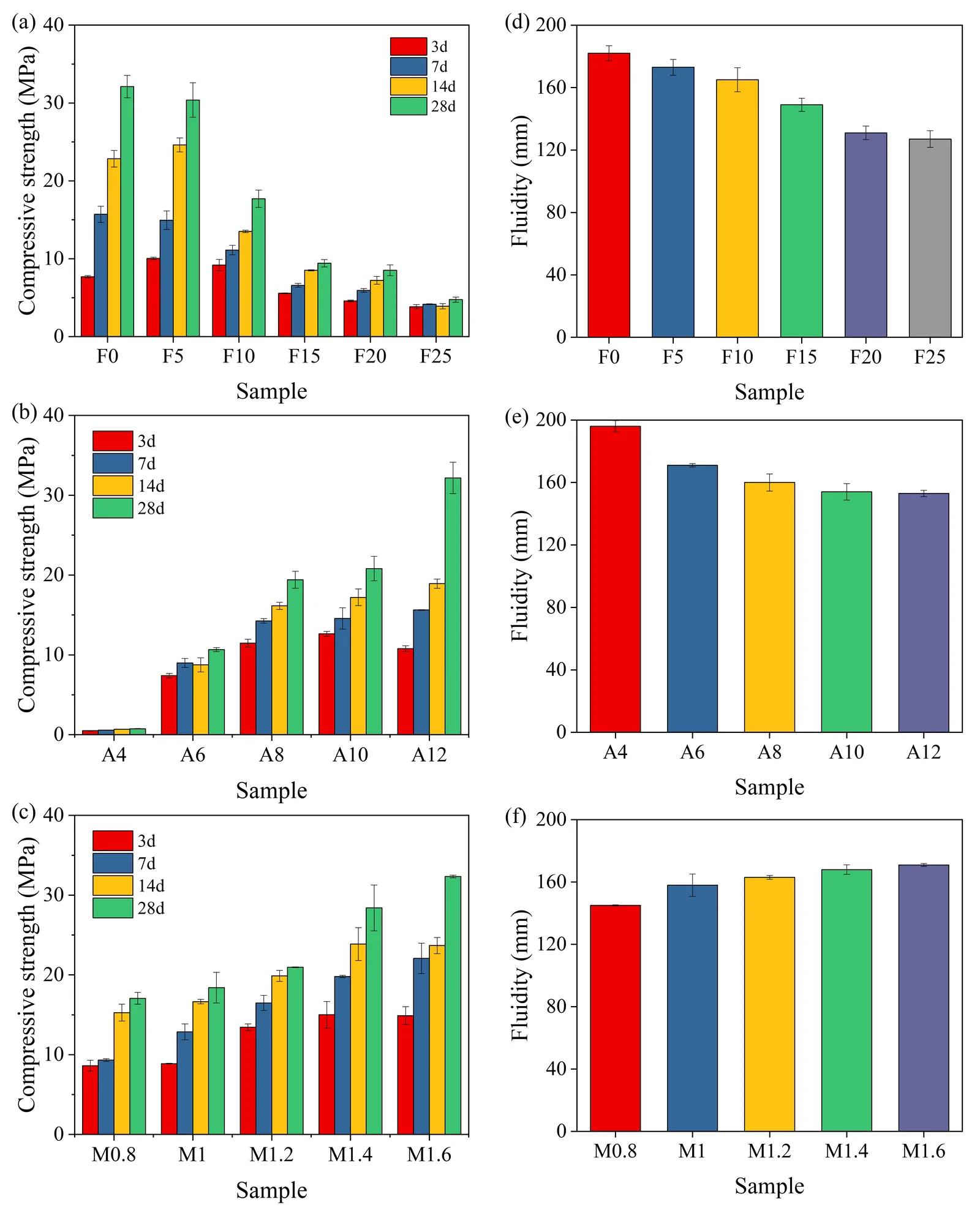
Changes in compressive strength (**a**–**c**) and fluidity (**d**–**f**) of the geopolymer.

The experimental setup involved a composite alkali activator dosage of 8%, an alkali activator modulus of 1.2, and a curing temperature of 50 °C. Figure 3 presented the macroscopic properties of geopolymers under various curing conditions. The control group (without CWFA) achieved a 28 d compressive strength of 32.11 MPa and a flowability of 182 mm. The introduction of CWFA resulted in a synchronous deterioration in both strength and fluidity. Although the compressive strength of all groups increased with curing age, the growth rate in high-content groups (F15–F25) was notably retarded. Furthermore, when the CWFA content exceeded 10%, the rate of strength degradation accelerated significantly. Specifically, the F5 group maintained the highest strength across all ages, reaching 32.61 MPa at 28 d. The compressive strength was 16.6 MPa with 10% addition of CWFA, and it further deteriorated to below 10 MPa with the 15–25% addition of CWFA. Notably, the F25 group suffered a drastic strength loss to 4.74 MPa, accompanied by a decrease in fluidity to 127 mm.

The performance deterioration was attributed to the multiple adverse effects of CWFA incorporation. Primarily, CWFA is characterized by a deficiency in reactive Si/Al species and a high content of impurities. As the dosage increased, the effective active components were diluted, resulting in insufficient gel formation to fill the pores. Concurrently, the accumulation of unreacted particles and interfacial defects disrupted the continuity of the geopolymer network. Additionally, the irregular morphology and high water absorption of CWFA particles exacerbated paste flocculation, leading to reduced fluidity and microstructural heterogeneity [40,41]. Although CWFA can provide some alkalinity, its inhibitory effect on the later-stage structural densification and strength development of the matrix predominates. Considering the balance between mechanical performance and waste utilization efficiency, 10% is identified as the acceptable addition of CWFA.

#### 3.3.2. Content of Alkali Activator

The experiments were conducted with 10% CWFA content, an activator modulus of 1.2, a curing temperature of 50 °C, and a water-to-binder ratio of 0.37. A dosage of only 4% alkali activator was insufficient to activate the system, as the inadequate alkali concentration failed to fully depolymerize the aluminosilicate precursors, hindering the formation of a continuous and stable gel skeleton [42]. Consequently, the 28 d compressive strength remained extremely low at 0.74 MPa. Increasing the dosage to 6% enhanced the dissolution capability of the precursors, leading to a noticeable improvement in strength, although it remained at a moderately low level. When the dosage was further raised to 8% and 10%, the compressive strength increased significantly, reaching 32.17 MPa at 28 d under the 10% regime. This indicated that a harmonious balance among raw material dissolution, ion migration, and gel polycondensation was achieved at this specific dosage. In contrast, while the 12% addition of alkali activator showed a slight increase in 28 d strength, its early and intermediate strength (3–14 d) was unsatisfactory. Furthermore, increasing the alkali activator dosage from 4% to 12% caused the flowability to drop progressively from 196 mm to 153 mm, as the higher alkali content intensified the cohesiveness of the slurry, thereby restricting its free fluidity [43]. Based on a comprehensive evaluation of strength development and fluidity, 10% was identified as the optimal alkali activator dosage. This proportion ensured sufficient excitation of the precursors and the formation of a dense microstructure while avoiding the flowability deterioration and formulation imbalance associated with excessive alkali content.

#### 3.3.3. Modulus of Alkali Activator

The experiments were conducted with fixed parameters: 10% CWFA content, 10% alkali activator dosage, a curing temperature of 50 °C, and a water-to-binder ratio of 0.37. As shown in Figure 3, as the alkali activator modulus increased from 0.8 to 1.6, the fluidity steadily improved from 145 mm to 171 mm, while the compressive strength exhibited a typical trend of rapid initial increase followed by stabilization. When the modulus increased from 0.8 to 1.4, the 28 d compressive strength rose significantly from 17.06 MPa to 30.41 MPa. Within this range, the concentrations of OH^−^ and soluble silicate species reached an optimal equilibrium, where the dissolution rates of Al and Si were well-matched, allowing monomers to fully condensate into a highly cross-linked three-dimensional network skeleton, thereby sustaining strength development [44]. However, upon further increasing the modulus to 1.6, the compressive strength only increased by a marginal 1.93 MPa, and the gain in flowability diminished, indicating that the reaction had approached saturation. Based on a comprehensive evaluation of strength and fluidity, a modulus of 1.4 was identified as the optimum, as it achieves the best compromise between high mechanical strength and favorable flowability.

### 3.4. Micro-Characteristics and Heavy Metal Migration Toxicity

**Figure 4:**
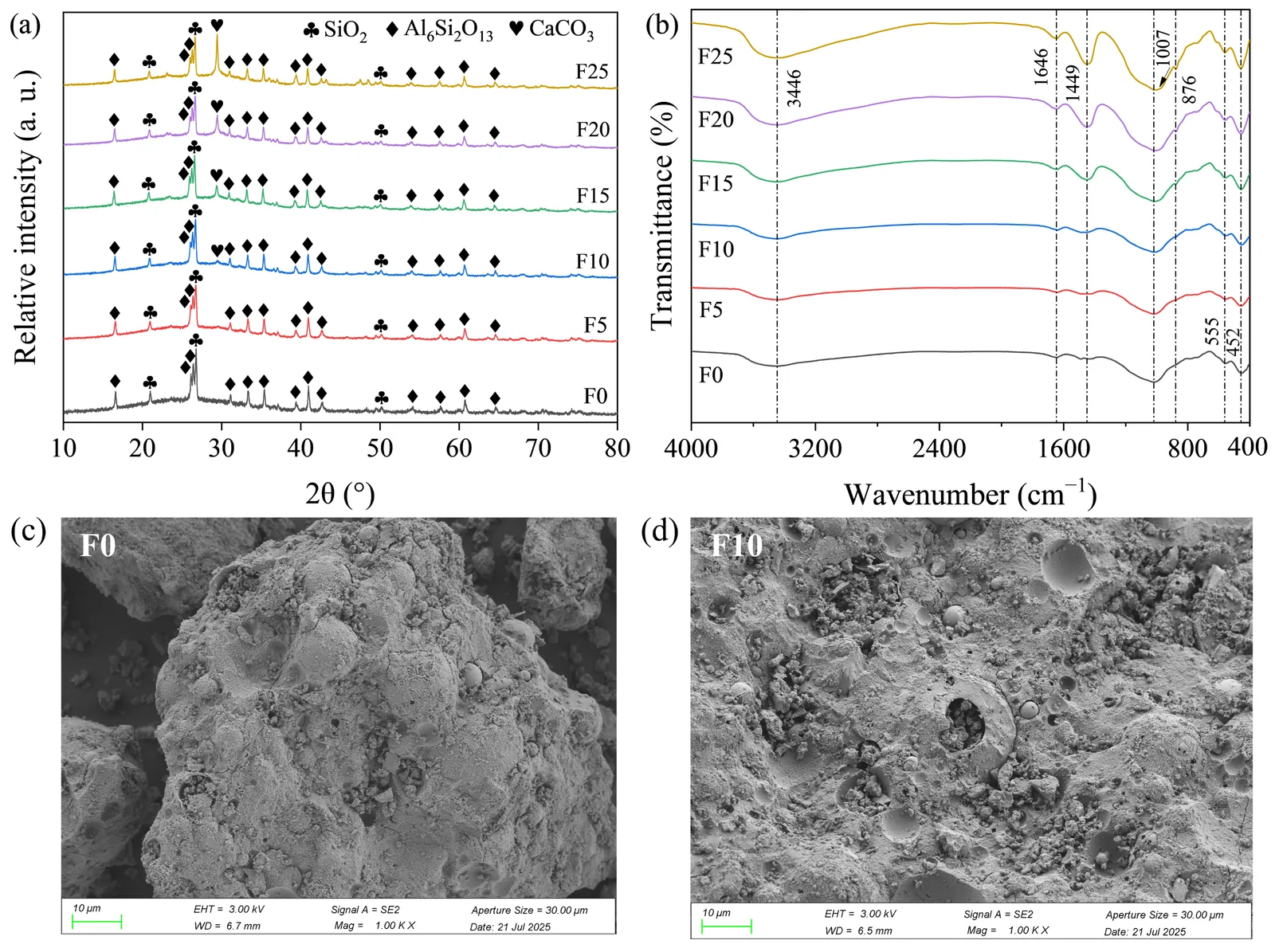
XRD (**a**), FTIR (**b**), and SEM (**c**,**d**) patterns of geopolymer.

Figure 4a presented the XRD patterns of geopolymer specimens with varying CWFA contents after 28 d of natural curing. All samples exhibited prominent diffraction peaks corresponding to SiO_2_ and Al_6_Si_2_O_13_, indicating that these phases were resistant to effective dissolution under ambient alkali activation conditions. As the CWFA content increased, the intensity of the CaCO_3_ diffraction peaks progressively enhanced. This suggested that the Ca components from CWFA were not entirely consumed to form amorphous gel phases, but also partially remained as crystalline residues or reprecipitated during the curing process. Although the incorporation of an appropriate amount of Ca facilitated the early nucleation and growth of gel products, driving the evolution of the gel system from a single N-A-S-H to a more complex C-(N)-A-S-H composite gel [13,45], the excessive crystalline residues and low-reactive mineral admixtures will compromise the integrity and continuity of the gel network, thereby corresponding to the observed deterioration in macroscopic strength [46].

Figure 4b displayed the FTIR spectra of geopolymer. The peaks around 3200–3600 cm^−1^ were caused by the stretching vibration of -OH, which can reflect the degree of reaction of the geopolymer [47]. The bending vibrational peak of H-O-H at 1645 cm^−1^ indicated that water is in geopolymer [48]. The bands near 1449 cm^−1^ represented the O-C-O stretching of carbonates, indicating the presence of carbonates when CWFA was introduced. These -OH groups may be associated with hydrated phases of alkaline aluminosilicate gels, and the water molecules are available deep in the pores of the matrix surface [49].

The vibration around 1019 cm^−1^ in F0 corresponded to the Si-O-T (T = Si/Al) vibration, indicating the formation of geopolymer [50]. Upon addition of 5% CWFA, the Si-O-T absorption peak almost did not shift. However, with further increase in CWFA content, this peak shifted in the opposite direction and even reached 1007 cm^−1^ at 25% CWFA. Additionally, as the CWFA content increased, the stretching vibration of OH around 3200–3600 cm^−1^ and the bending vibration peak of H-O-H at 1646 cm^−1^ also intensified. All these observations suggest that incorporating CWFA altered the coordination environment of Si/Al in the gel, promoting structural reorganization of the gel network [50]. Notably, with increasing CWFA content, both the broad O-H stretching vibration band at 3200–3600 cm^−1^ and the H-O-H bending vibration peak near 1646 cm^−1^ showed synchronized enhancement. This was primarily attributed to reduced reaction extent at high CWFA levels, leading to increased matrix porosity and enhanced adsorption capacity for environmental water [51]. This will lead to residual unreacted particles, increased porosity, and the development of interfacial defects, thereby reducing geopolymer strength.

Besides, under the action of composite chemical activators, the abundant Ca component in CWFA synergistically dissolved with Si/Al components, triggering coupled polymerization and hydration reactions. This process generated amorphous Ca-bearing silicate (C-A-S-H/C-S-H) gels and portlandite, while some Ca was successfully incorporated into the three-dimensional network. These formed gel products effectively fill the interstitial voids between CWFA and hollow glass microspheres, and tightly cement the unreacted inert particles, thereby constructing a dense binary geopolymer matrix [52]. This microstructural evolution reasonably explained the significant enhancement of Si-O-Al and Si-O-Si (near 555 and 452 cm^−1^) vibration peaks in the FTIR spectra after the incorporation of a small amount of CWFA with insufficient Si/Al content [49,53], and the fact that the compressive strength of the 5% CWFA sample (F5) remained almost unchanged compared to the reference (F0) at 3–14 d, even exhibiting a slight early-age strength advantage.

Figure 4c,d illustrated the microstructures of F0 and F10, respectively. The pure CFA geopolymer (F0) showed a compact and continuous matrix with few pores, which indicated that the 50 °C curing temperature for 1 day was insufficient to fully activate the precursor and achieve complete dissolution of the fly ash particles. In comparison, the F10 sample exhibited a more porous and loose structure with evident microcracks and unreacted particles. The incorporation of 10% CWFA introduced soluble chlorides and inert crystalline impurities, which partially displaced reactive Si/Al-rich precursors and thereby lowered the effective dissolved Si/Al pool. Concurrently, high Cl^-^ activity may also retard the dissolution-polycondensation stage and divert available Ca/Al into Friedel’s salt rather than into C-(N-)A-S-H cross-links [54,55,56]. These effects collectively weakened gel interconnectivity, enlarged the interfacial transition zone around unreacted CWFA particles and promoted structural loosening [57], which was also evidenced by the observed decrease in compressive strength for the F10 group. It should also be noted that, such microcrack features may also be partly accentuated by drying, vacuum, and electron-beam effects during SEM observation.

The heavy metals leaching toxicity of MSWIFA and the geopolymer incorporating 10% CWFA (F10) was tested to evaluate the solidification/stabilization efficiency of the geopolymer matrix under different curing time, with the results presented in Table 3. The leaching concentrations of Cd, Pb, and Zn in the raw MSWIFA were far higher than the regulatory limits specified in the Standard for Pollution Control on the Landfill Site of Municipal Solid Waste GB 16889-2024 [58], indicating a significant environmental risk. After geopolymerization, the leaching concentrations of all detected heavy metals in the F10 sample decreased significantly and continued to decline with prolonged curing ages. This reduction was attributed to the dense aluminosilicate gel network formed during alkali activation, which effectively immobilizes heavy metals through physical encapsulation, surface adsorption, and chemical bonding [59]. Specifically, the leaching concentrations of As, Cu, Pb, and Zn fell below the method detection limit by 3 d. By 28 d, the migration risks of all heavy metals were effectively controlled. However, Cd and Ni exhibited relatively weaker immobilization efficiencies, as their concentrations remained detectable, with Ni showing only a 95.65% reduction. These discrepancies in leaching behavior are primarily related to the original speciation of the metals in the MSWIFA and CWFA and their specific interactions with the pore structure and local chemical environment within the geopolymer matrix [12,60].

### 3.5. Effect of Ca(OH)_2_ on the Geopolymer Properties

With increasing Ca(OH)_2_ content from 0% to 10%, the compressive strength of the geopolymers exhibited an overall trend of initial enhancement followed by a slight decline. The control group (CH0) consistently yielded the lowest strengths across all curing ages, indicating that although the system could develop a certain degree of strength relying solely on its inherent reactive aluminosilicate sources, the contribution of extended curing to strength gain was relatively limited. Increasing the Ca(OH)_2_ dosage to 2.5% and 5% resulted in a marked enhancement in compressive strength. This improvement suggests that moderate Ca(OH)_2_ accelerated the cleavage of Si-O and Al-O bonds, thereby facilitating the dissolution of precursors and the subsequent formation of gel phases. Furthermore, the incorporated Ca^2+^ ions can substitute for Na^+^ within the N-A-S-H gel framework, promoting the precipitation of C-A-S-H or (N, C)-A-S-H gels [61]. Consequently, this leads to a synergistic microstructure characterized by the mutual interlocking and co-growth of dual gel networks. A further increase to 7.5% resulted in optimal strength development at all tested ages, with the 28 d compressive strength peaking at 53.33 MPa. However, raising the dosage to 10% failed to yield additional strength benefits, with the 28 d strength decreasing to 46.28 MPa.

Concurrently, the fluidity of the fresh paste decreased progressively with higher Ca(OH)_2_ loadings. This reduction can be attributed to the high hydrophilicity of Ca(OH)_2_, which elevated the overall water demand of the binder system. Furthermore, the fine Ca(OH)_2_ powders physically filled the interstitial voids within the slurry, reducing the free space available for flow and resulting in a stiffer consistency. Additionally, the accelerated formation of C-A-S-H/C-S-H gels induced by excess Ca(OH)_2_ rapidly consumed free water, thereby causing a swift loss of fluidity.

**Figure 5:**
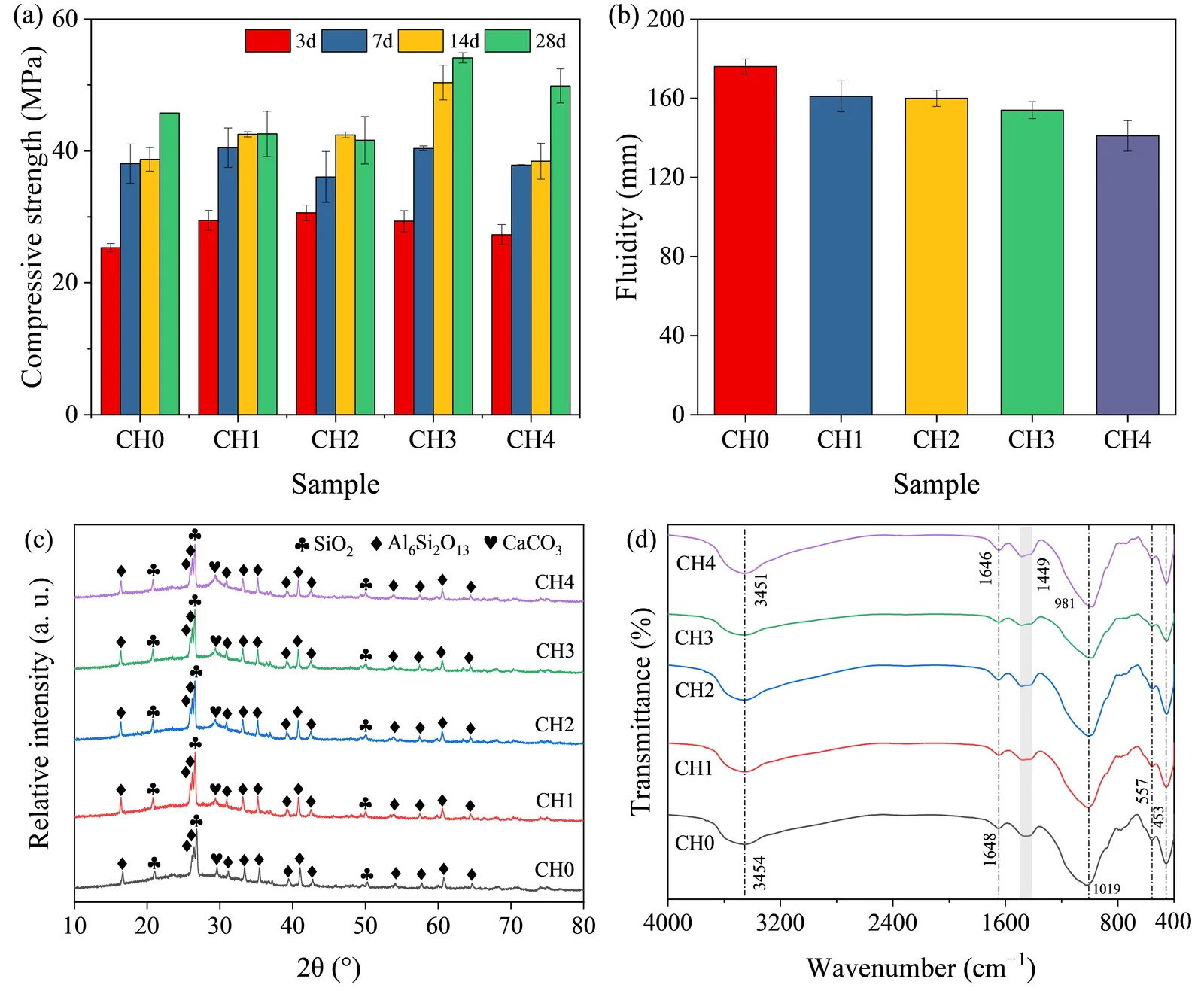
Characteristics of geopolymer with Ca(OH)_2_ addition: (**a**) compressive strength, (**b**) fluidity, (**c**) XRD, and (**d**) FTIR.

As shown in Figure 5c, the phase composition varied significantly with increasing Ca(OH)_2_ content. The control group (CH0) exhibited diffraction peaks corresponding to Al_6_Si_2_O_13_ and SiO_2_. Notably, the absence of a discernible amorphous halo in the 20–35° range indicated limited geopolymerization activity and negligible formation of amorphous gel phases. With increasing Ca(OH)_2_ addition, a progressive uplift of the baseline beneath the CaCO_3_ diffraction peaks was observed. At 10% Ca(OH)_2_ (CH4) addition, the CaCO_3_ peak at 29.4° intensified markedly, superimposed on a prominent broad hump indicative of an extensive amorphous background. This distinct baseline elevation resulted from the superposition of intense XRD by the newly formed amorphous gel phases and the sharp diffraction signals from crystalline CaCO_3_. These features confirmed that while excess Ca(OH)_2_ effectively promoted precursor dissolution and gel precipitation, the promotional effect of Ca(OH)_2_ had reached a saturation point, beyond which excessive Ca(OH)_2_ induced excessive precipitation of crystalline Ca-rich phases (e.g., calcite) and accelerated carbonation, which introduced microstructural discontinuities and consequently led to a slight deterioration in compressive strength [46]. This observation correlated well with the slight decrease in 28 d compressive strength observed for CH4 compared to the optimum performance of CH3.

Figure 5d illustrated the evolution of FTIR spectra for geopolymers with different Ca(OH)_2_ additions after a curing period of 28 d. With increasing Ca(OH)_2_ dosage from CH0 to CH4, the FTIR spectra exhibited systematic evolution. The asymmetric stretching vibration band of Si-O-T (T = Si, Al) shifted continuously from 1017.63 cm^−1^ to 979.02 cm^−1^ within the 979–1018 cm^−1^ region, accompanied by an initial increase followed by stabilization in both bandwidth and absorbance intensity, peaking at CH2. This phenomenon was attributed to the dual role of Ca^2+^. Firstly, it can act as a co-activator to enhance the dissolution of aluminosilicate phases by OH^-^, thereby promoting the depolymerization of [SiO_4_]/[AlO_4_] units. Secondly, it can substitute Na^+^ in N-A-S-H gels or directly participate in polycondensation to form Ca-rich C-A-S-H gels. The increased incorporation of Ca^2+^ elevated the substitution degree of Si^4+^ by Al^3+^, leading to elongated Si-O(Al) bond lengths and reduced bond energy, which accounted for the observed redshift [62]. The stabilized peak intensity beyond CH2 suggested that excessive Ca^2+^ promoted the over-precipitation of CaCO_3_ and impeded the continuous development of the three-dimensional gel network.

The stretching vibration of -OH at 3454 cm^−1^ and the bending vibrational peak of H-O-H at 1648 cm^−1^ exhibited a general trend of increasing intensity and slight red-shift with higher Ca(OH)_2_ dosages, although a transient shift was observed at CH2 due to localized carbonation. This indicated an increase in bound water content within the gel structure, corroborating the enhanced formation of geopolymeric products, which was consistent with previous observations upon CWFA incorporation. Concurrently, the progressive intensification of the carbonate band (1420–1484 cm^−1^) confirmed that elevated free Ca^2+^ levels accelerated carbonation during curing. In contrast, the Si-O-Al and Si-O-Si bending vibrations near 557 cm^−1^ and 453 cm^−1^ showed negligible changes in either position or intensity, suggesting that Ca(OH)_2_ did not alter the fundamental framework of the silicate tetrahedra.

**Figure 6:**
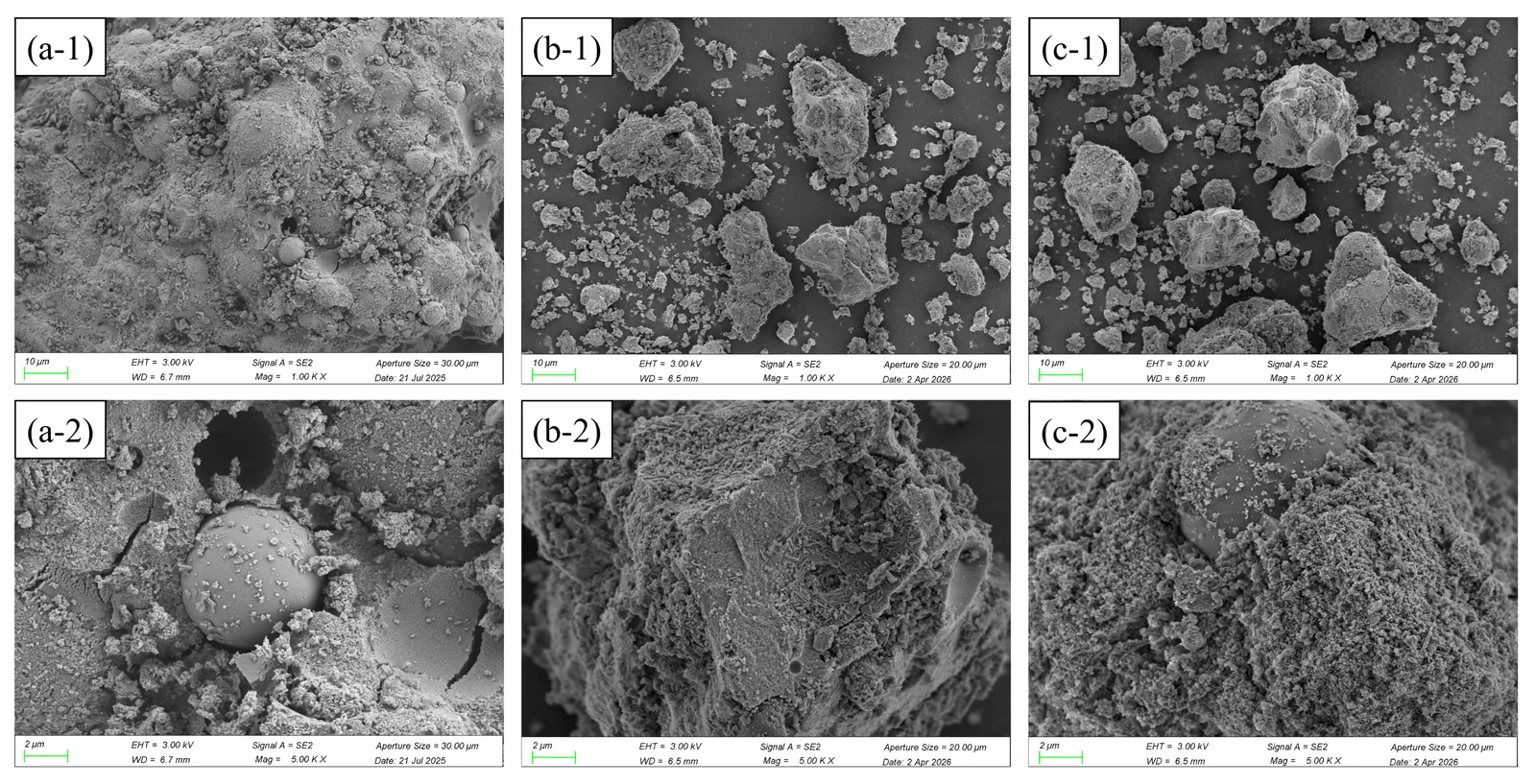
Surface morphology of geopolymer with Ca(OH)_2_ addition: (**a-1**) CH0–1000 magnification, (**a-2**) CH0–6000 magnification, (**b-1**) CH2–1000 magnification, (**b-2**) CH2–6000 magnification, (**c-1**) CH4–1000 magnification, (**c-2**) CH4–6000 magnification.

The SEM results in Figure 6 revealed that the CH0 group exhibited a relatively loose matrix, characterized by widely dispersed spherical particles with distinct boundaries and weak inter-particle bonding, along with localized pores and microcracks. As the Ca(OH)_2_ content increased to the CH2 group, a continuous and dense cementitious phase gradually developed on the sample surface. High-magnification imaging further revealed an intertwined network of short fibrous and massive/flocculent structures within the matrix, especially in the CH4 sample, which may be attributed to C-A-S-H and N-A-S-H gels, respectively [63]. These findings suggest that an appropriate dosage of Ca(OH)_2_ effectively promoted the depolymerization-polycondensation of [Si/Al–O] bonds, significantly enhancing both gel yield and cross-linking density. In contrast, the CH4 group displayed pronounced interfacial gaps and was enriched with fine particulate and flocculent precipitates, primarily attributed to the excessive formation of crystalline phases and accelerated carbonation. The dispersion of these crystalline phases within the gel network disrupted structural continuity, demonstrating that excessive Ca(OH)_2_ incorporation impeded the normal development of the cementitious microstructure.

Ca(OH)_2_ incorporation markedly enhanced heavy metal immobilization within the geopolymer matrix. As summarized in Table 4, As, Cr, Cu, Pb, and Zn remained undetectable across all specimens, comfortably meeting GB 16889-2024 thresholds [58]. Notably, Cd and Ni leaching declined progressively from CH0 to CH3 (7.5% Ca(OH)_2_), reaching minima of 0.01 mg/L and ND, respectively. This exceptional retention stems from the Ca^2+^-induced transformation of the gel architecture: moderate Ca(OH)_2_ promoted the coexistence of C-(N)-A-S-H and N-A-S-H networks, where Ca^2+^ substituted interlayer Na^+^ and precipitated as Ca-bearing gels that possess higher affinity for divalent cations via isomorphic substitution and stronger electrostatic confinement [61,62]. Simultaneously, the refined pore structure reduced diffusional pathways for leachates. At CH4 (10%), Cd and Ni rebounded slightly to 0.03 mg/L and 0.01 mg/L. This may be attributed to the microstructural discontinuities caused by the excessive formation of crystalline phases and accelerated carbonation, which compromised the encapsulation efficiency of the geopolymer matrix [26]. Nonetheless, all values remained orders of magnitude below regulatory limits, underscoring the efficacy of Ca-mediated gel engineering in securing environmental safety.

### 3.6. Discussion on This MSWI Utilization Technology

The integrated pretreatment combining calcination at 350 °C with subsequent water washing effectively destroyed refractory dioxins and removed chloride salts, while ensuring the superior immobilization of heavy metals during geopolymerization. These attributes render the process suitable for producing greenconstruction materials.

Compared with energy-intensive vitrification or conventional cement solidification, this co-processing route offers a more sustainable alternative. Nevertheless, the moderate waste-loading rate still remains a challenge for large-scale deployment, as incorporating excessive CWFA will significantly compromise compressive strength and fluidity. This stems from three factors: (i) dilution of reactive Si/Al precursors, leading to insufficient gel formation; (ii) high water absorption and irregular morphology of CWFA particles, which increase water demand and reduce fluidity; and (iii) accumulation of unreacted crystalline residues (e.g., CaCO_3_) and interfacial defects. These microstructural discontinuities disrupt the three-dimensional network, causing a drastic strength loss and limiting the practical waste-loading capacity too.

To overcome these barriers and increase the MSWIFA incorporation limit without sacrificing mechanical or environmental performance, future research should focus on (i) introducing highly reactive aluminosilicate supplements (e.g., metakaolin) to compensate for Si/Al deficiency and boost gel nucleation; (ii) refining curing method (e.g., microwave or thermal curing) to accelerate reaction kinetics; and (iii) exploring hybrid pretreatment methods to further enhance MSWIFA reactivity.

## 4. Conclusions

In this work, the properties of geopolymers prepared by the co-processing of MSWIFA and CFA were studied, and the influence of exogenous addition of Ca(OH)_2_ was also investigated. The experimental results of this study can be summarized as follows:

(1) The water washing pretreatment effectively eliminated soluble NaCl and KCl. The low-temperature calcination at 350 °C attained a 98.92% PCDD/Fs TEQ-removal efficiency, which satisfied the toxicity threshold required by HJ 1134-2020 [36]. The systematic optimization confirmed that a liquid–solid ratio of 8, a single washing duration of 70 min, and three repeated washing cycles delivered balanced dechlorination efficiency and water consumption, simultaneously removing dioxins and soluble chloride contaminants to eliminate adverse impacts on geopolymer fluidity and long-term durability.
(2) The compressive strength and fluidity of the geopolymers were strongly governed by the CWFA dosage, alkali activator content, and modulus. While 10% CWFA alone did not contribute positively to mechanical performance, a formulation comprised 10% CWFA replacement, 10% alkali activator content, and a modulus of 1.4 was accepted in term of maximizing MSWI utilization. The incorporation of 7.5% Ca(OH)_2_ under alkaline activation accelerated aluminosilicate dissolution and promoted the coexistence of N-A-S-H and C-(N)-A-S-H gels, thereby increasing the 28 d compressive strength to 53.33 MPa. However, the overdosed Ca(OH)_2_ accumulated crystalline CaCO_3_ to form microstructural defects and reduced the compressive strength of geopolymer.
(3) The geopolymer matrix stabilized heavy metals via physical encapsulation, surface adsorption, and chemical bonding. After 28 d curing, nearly all heavy metal leaching concentrations met GB 16889-2024 limits. This combined detoxificationwashing pretreatment and calcium modification strategy provided a feasible technical pathway for harmless and high-value recycling of MSWIFA.

## Figures and Tables

**Table 1:** Chemical composition of experimental materials (wt%).

	CaO	Cl	Na_2_O	SO_3_	K_2_O	SiO_2_	Fe_2_O_3_	TiO_2_	MgO	Al_2_O_3_	Others
MSWIFA	35.56	26.93	12.52	10.83	7.41	3.11	0.91	-	0.69	0.4	1.64
CFA	6.19	-	1.17	3.9	0.51	41.06	3.35	2.2	0.86	39.84	0.92

**Table 2:** Mix proportion for experiment.

No.	CWFA (wt%)	CFA (wt%)	NaOH (wt%)	Ca(OH)_2_ (wt%)	n(SiO_2_/Na_2_O)	W/B
F0	0	100	8	0	1.2	0.33
F5	5	95	8	0	1.2	0.35
F10	10	90	8	0	1.2	0.38
F15	15	85	8	0	1.2	0.41
F20	20	80	8	0	1.2	0.44
F25	25	75	8	0	1.2	0.47
A4	10	90	4	0	1.2	0.37
A6	10	90	6	0	1.2	0.37
A8	10	90	8	0	1.2	0.37
A10	10	90	10	0	1.2	0.37
A12	10	90	12	0	1.2	0.37
M0.8	10	90	10	0	0.8	0.37
M1	10	90	10	0	1.0	0.37
M1.2	10	90	10	0	1.2	0.37
M1.4	10	90	10	0	1.4	0.37
M1.6	10	90	10	0	1.6	0.37
CH0	10	90	10	0	1.4	0.35
CH1	10	90	10	2.5	1.4	0.38
CH2	10	90	10	5	1.4	0.41
CH3	10	90	10	7.5	1.4	0.44
CH4	10	90	10	10	1.4	0.47

**Table 3:** Leaching toxicity of geopolymer F10 under different curing time (mg/L).

	MSWIFA	F10-3 d	F10-7 d	F10-14 d	F10-28 d	GB 16889-2024 [58]
As	0.06	ND	ND	ND	ND	0.3
Cd	7.49	0.09	0.08	0.06	0.04	0.15
Cr	0.49	0.02	0.01	ND	ND	4.5
Cu	7.42	ND	ND	ND	ND	40
Ni	0.42	0.03	0.03	0.03	0.02	0.5
Pb	19.19	ND	ND	ND	ND	0.25
Zn	151.59	ND	ND	ND	ND	100

ND: Not Detected.

**Table 4:** Leaching toxicity of geopolymer with Ca(OH)_2_ addition (mg/L).

	CH0	CH1	CH2	CH3	CH4	GB 16889-2024 [58]
As	ND	ND	ND	ND	ND	0.3
Cd	0.04	0.03	0.02	0.01	0.03	0.15
Cr	ND	ND	ND	ND	ND	4.5
Cu	ND	ND	ND	ND	ND	40
Ni	0.02	0.01	0.01	ND	0.01	0.5
Pb	ND	ND	ND	ND	ND	0.25
Zn	ND	ND	ND	ND	ND	100

ND: Not Detected.

## Data Availability

The original contributions presented in this study are included in the article. Further inquiries can be directed to the corresponding authors.

## Abbreviations

The following abbreviations are used in this manuscript:

MSWIFA	municipal solid waste incineration fly ash
CWFA	calcined and washed municipal solid waste incineration fly ash
n(SiO_2_/Na_2_O)	molar ratio of SiO_2_ to Na_2_O
W/B	the mass ratio of exterior water and solid materials
